# Role of Biomolecules in Osteoclasts and Their Therapeutic Potential for Osteoporosis

**DOI:** 10.3390/biom11050747

**Published:** 2021-05-17

**Authors:** Xin Zhao, Suryaji Patil, Fang Xu, Xiao Lin, Airong Qian

**Affiliations:** 1 School of Pharmacy, Shaanxi Institute of International Trade & Commerce, Xi’an 712046, China; zx2680271@126.com; 2 Xi’an Key Laboratory of Special Medicine and Health Engineering, Key Lab for Space Biosciences and Biotechnology, Research Center for Special Medicine and Health Systems Engineering, NPU-UAB Joint Laboratory for Bone Metabolism, School of Life Sciences, Northwestern Polytechnical University, Xi’an 710072, China; suryajip@mail.nwpu.edu.cn (S.P.); xufang@mail.nwpu.edu.cn (F.X.); linxiao@nwpu.edu.cn (X.L.)

**Keywords:** osteoclasts (OCs), bone resorption, osteoporosis, therapeutics, gene therapy

## Abstract

Osteoclasts (OCs) are important cells that are involved in the regulation of bone metabolism and are mainly responsible for coordinating bone resorption with bone formation to regulate bone remodeling. The imbalance between bone resorption and formation significantly affects bone metabolism. When the activity of osteoclasts exceeds the osteoblasts, it results in a condition called osteoporosis, which is characterized by reduced bone microarchitecture, decreased bone mass, and increased occurrences of fracture. Molecules, including transcription factors, proteins, hormones, nucleic acids, such as non-coding RNAs, play an important role in osteoclast proliferation, differentiation, and function. In this review, we have highlighted the role of these molecules in osteoclasts regulation and osteoporosis. The developed therapeutics targeting these molecules for the treatment of osteoporosis in recent years have also been discussed with challenges faced in clinical application.

## 1. Introduction

Bone remodeling constitutes a synchronized action of osteoclasts and osteoblasts and involves activation of osteoclasts, resorption of old bone by osteoclasts, reversal, formation of new bone by osteoblasts, and termination [1]. The systemic regulators, such as hormones, including sex hormones, parathyroid hormone, and calcitonin (CT), as well as local factors, including growth factors and cytokines, regulate the process of bone remodeling [2]. It is known that osteoblasts and osteoclasts communicate via cytokines, extracellular matrix interaction, and directly through cell contract to regulate each other’s formation, differentiation, or apoptosis through multiple pathways, transcription factors, and proteins [3]. Numerous studies have reported the role of osteoblasts in osteoporosis, but seldom reported the role of osteoclasts. The bone resorption is mainly carried out by osteoclasts. These cells have unique morphological, as well as phenotypic characteristics, such as more than one nucleus and tartrate-resistant acid phosphatase (TRAP) and the calcitonin receptor expression. These are found on the surface of the bone and are believed to be produced in the bone marrow [4]. Hematopoietic progenitor cells (HPCs), as well as signals from the microenvironment, such as macrophage colony-stimulating factor (M-CSF) and receptor activator of nuclear factor kappa B (NF-κB) ligand (RANKL), are needed for osteoclast formation [5]. Any disruption in the bone remodeling process or imbalance between bone resorption and formation can cause bone diseases, including osteoporosis. Osteoporosis, the most common and multifactorial bone disease, is characterized by reduced bone mass, weakened microarchitecture, and increased risk of fractures [6]. It affects over 200 million people worldwide. Despite being most prevalent in postmenopausal women, one in four men is at risk of suffering an osteoporotic fracture, making it one of the serious diseases that need pressing attention [7,8]. Notably, higher osteoclastic bone resorption compared with bone formation contributes significantly to the development of osteoporosis [9]. Therefore, it is critical to understand the factors that regulate osteoclast function to explore the novel therapeutics targeting osteoclasts. This review summarizes the factors affecting osteoclast function well as therapeutics that have been developed to target osteoclasts to help in developing therapeutic agents.

## 2. The Role of Molecules in Osteoclast Regulation

### 2.1. Cytokines

**Figure 1:**
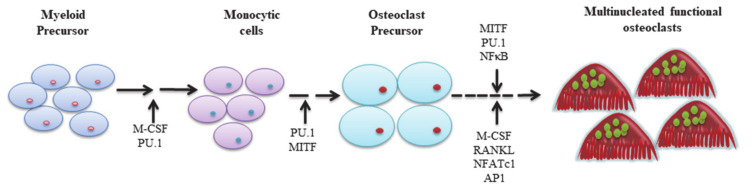
Factors affecting osteoclast differentiation.

Osteoclasts are derived from pluripotent hematopoietic stem cells that differentiate into myeloid stem cells. Myeloid stem cells differentiate into four kinds of cells, namely megakaryocytes, granulocytes, monocytes/macrophages, and osteoclasts. Myelocytic series is differentiated from stem cells to form the granulocyte-macrophage colony-forming unit (CFU-GM), which further differentiate into osteoclast precursor and (pre-OC) and eventually form mature osteoclasts. Moreover, various cytokines are involved in this process, such as PU.1, M-CSF, c-Fos, CCAAT/enhancer-binding protein α (C/EBPα), RANK, NFATc1, and microphthalmia-associated transcription factor (MITF) [10,11] (Figure 1).

#### 2.1.1. PU.1

PU.1 is predominantly expressed in hematopoietic cells, such as B cells, macrophages, and neutrophils, and is a member of the Ets family protein [12]. Its expression is proportional to the rate of macrophage differentiation to the osteoclast, and PU.1 deficiency can halt the development of osteoclasts, as well as macrophages [13]. Generally, PU.1 promotes the expression of nuclear factor of activated T-cells cytoplasmic 1 (NFATc1) and upregulates osteoclast-specific gene expression by directly binding to the NFATc1 promoter [14]. PU.1 regulates RANKL-induced cathepsin K (CTSK) expression by interacting with NFATc1, p38 mitogen-activated protein kinase (MAPK), and MITF, to promote osteoclastogenesis and osteoclast differentiation [15,16]. During the differentiation of bone marrow-derived macrophages (BMMs) to osteoclast, PU.1 shifts its transcription partner from interferon regulatory factor (IRF) 8 to NFATc1 under the influence of RANKL and controls cell-type-specific gene expression during osteoclastogenesis [17].

#### 2.1.2. M-CSF

M-CSF is essential for the differentiation of monocyte-derived macrophages [18], and regulates various tissue macrophage and monocyte populations [19]. Proverbially, it is well established that the formation, survival, and migration of osteoclasts are also governed by M-CSF [20]. Besides, under the stimulus of M-CSF, granulocyte/macrophage colony-forming cells (GM-CFCs) proliferate and differentiate into preosteoclasts that fuse to form mature osteoclasts [21]. M-CSF can also promote mature OC resorbing activity through RANKL-induced activation of Finkel-Biskis-Jinkins osteosarcoma (c-Fos) and extracellular signal-regulated kinase (ERK) 1/2 phosphorylation [22]. It has demonstrated that the mutation in M-CSF can significantly reduce the number of osteoclasts, as well as macrophages in vivo [23]. M-CSF produced by mesenchymal cells promotes survival, proliferation, and differentiation of osteoclast precursor cells through its receptor, c-Fms. Specifically, M-CSF binding to c-Fms and/or αvβ3 engages adapter proteins and cytosolic kinases to activate intracellular signals, Syk and Vav3, to influence survival, function [24], and differentiation of osteoclasts [25]. M-CSF/RANKL signaling, regulated by an E3 ubiquitin ligase and Notch inhibitor, Ligand of numb binding protein X 2 (LNX2), also induces macrophages proliferation and osteoclasts survival by activating ERK and the phosphoinositide-3-kinase (PI3K)/AKT pathways [26].

#### 2.1.3. c-Fos

Activator protein-1 (AP-1) family proteins regulate proliferation, differentiation, and developmental transformation of many cells, including osteoclasts. c-Fos, a component of AP-1, is an important factor for osteoclast differentiation [27], and positively regulates osteoclastogenesis. c-Fos inhibits differentiation, as well as activation of mononuclear phagocytes and dendritic cell development [28,29]. The absence of c-Fos leads to the deficiency of NFATc1 and blocks osteoclast differentiation. Therefore, inhibition of c-Fos can be considered a promising mechanism for suppressing osteoclast differentiation [29].

#### 2.1.4. C/EBPα

C/EBPα is a key cis-acting regulatory element of the promoter of osteoclast-specific CTSK that plays a vital role during the commitment of osteoclast lineage [30]. The lack of C/EBPα impairs osteoclastogenesis and leads to osteopetrosis. C/EBPα in monocyte/macrophage cells stimulates the expression of receptor activator of NF-κB (RANK), NFATc1, c-Fos, and CTSK, and promotes monocyte/macrophage cell differentiation to osteoclast-like cells [30]. Furthermore, C/EBPα regulates osteoclast activity by inducing specific gene expression of osteoclasts, stimulating extracellular acidification [11], as well as terminal differentiation, activation, and function of osteoclast by directly regulating NFATc1 [31]. It is indispensable in stimulating extracellular acidification and regulating cell survival through RANKL-induced Akt activation, to ensure OC survival [32]. The exclusive cytoplasmic domain of RANK labeled as ^535^IVVY^538^ motif is essential for osteoclast differentiation and is regulated by C/EBPα to induce osteoclasts differentiation [33].

#### 2.1.5. RANK/RANKL/Osteoprotegerin (OPG)

RANK and RANKL, a pair of a receptor and a ligand belonging to the tumor necrosis factor (TNF) family, are the essential regulators of osteoclast activity [34]. RANKL is a type II transmembrane protein expressed in osteoblasts, activated T cells, lymph nodes, thymus, breast, and lung cells as membrane-bound and a secreted protein [35]. RANK is a type I transmembrane protein member of the TNF receptor (TNFR) superfamily through which RANKL transmits a signal and is highly expressed on the membrane of osteoclast progenitor, mature osteoclasts, dendritic cells, and mammary glands [36].

RANKL stimulates RANK to employ TNF receptor-associated factor 6, (TRAF6), which in turn activates PI3K and MAPK. This stimulates NFATc1, c-Fos, and NF-κB to translocate to the nucleus and become activated [37]. NFATc1 and c-Fos promote transcription of the genes required for osteoclastogenesis [38]. OPG, produced by osteoblasts, regulates the binding of RANKL to RANK and inhibits transcription of osteoclastogenesis genes, osteoclast differentiation, and activation of mature osteoclasts, ultimately lowering excessive bone resorption. However, excessive OPG can cause severe osteopetrosis due to a reduced number of mature osteoclasts, while knockout leads to osteoporosis [39,40,41,42].

Interestingly, the binding of osteoclasts-secreted vesicular RANK to RANKL promotes bone formation through RANKL reverse signaling by activating Runx2 [43]. In 1998, a RANKL/TNF-related activation-induced cytokine (TRANCE) identical factor, osteoclast differentiation factor (OCIF), was identified as an osteoprotegerin ligand (OPGL) that could stimulate osteoclast progenitor cells to differentiate to mature osteoclasts [44], and acts as a positive factor for osteoclast differentiation and regulates T cell and dendritic cell interactions [45]. In addition, RANK/TRAF regulates osteoclasts formation and activation through JNK/AP-1, an inhibitor of NF-κB kinase (IKK)/NF-κB, C-MYC, calcineurin/NFATc1, Src, and MKK6/p38/MITF [41]. NFATc1 is an important transcription factor that plays a vital role in regulating the expression of tartrate-resistant acid phosphatase (TRAP) and osteoclast-associated receptor (OSCAR), and its expression is prompted and acetylated by RANKL during osteoclastogenesis [46]. Additionally, leucine-rich repeat-containing G-protein-coupled receptor 4 (LGR4)/GPR48) can also compete with RANK for binding to RANKL and weaken downstream signaling of osteoclast activation and differentiation [47].

#### 2.1.6. NFATc1

NFATc1 and NFATc2 are essential factors for the commitment of cell lineage, as well as osteoclasts differentiation [48,49]. Strong induction of NFATc1 is known as autoamplification of NFATc1 and is dependent on calcium signaling of immunoglobulin-like receptors associated with immunoreceptor tyrosine-based activation motif (ITAM)-harboring adaptors. Moreover, calcium signaling also initiates phosphatase calcineurin and stimulates NFATc movement into the nucleus, where it forms complexes with other proteins on DNA and enhances osteoclastogenesis [50,51]. In addition, the AP-1 complex containing c-Fos can bind to NFATc1 to achieve strong induction of NFATc1 [48], and the expression of osteoclast-specific genes, such as TRAP [52], calcitonin receptor [49], cathepsin k [15], and β3 integrin are directly regulated by NFATc1 [53]. The report showed that NFATc1-deficient embryonic stem cells can not differentiate into osteoclasts. In the absence of RANKL, ectopic expression of NFATc1 prompts bone marrow-derived precursor cells to differentiate to osteoclasts [49].

#### 2.1.7. MITF

MITF is an essential factor required for terminal differentiation of osteoclast and targets signaling pathways involving CSF-1 and RANKL. MITF wields its regulatory effects by associating itself with cofactors, such as 14-3-3 and Cdc25C-associated kinase (C-TAK)-1 to translocate from the cytoplasm to the nucleus to regulate monocytic precursor differentiation [54,55]. Moreover, MITF is phosphorylated by p38 MAPK to form a trimeric complex with proto-oncogene fused in sarcoma (FUS) and chromatin remodeling ATPase BRG1 during osteoclast differentiation [56] MITF, myocyte enhancer factor 2 (MEF2), and NFATc1 activate V-ATPase proton pump d2 promoter and cathepsin K promoter and stimulates RANKL-induced osteoclastogenesis in osteoclast precursor cells [57,58].

### 2.2. Hormone

#### 2.2.1. Estrogen

Estrogen plays an important role in bone metabolism by applying a protective effect on bone [59]. Estrogen employs different factors, such as hypoxia-inducible factor 1α(HIF1α), vascular endothelial growth factor (VEGF), interleukin (IL)-1, IL-2, IL-6, NF-κB, RANKL, to exert an inhibitory effect on osteoclasts. At the cellular level, estrogen inhibits osteoclast differentiation by decreasing their number, probably through some cytokines, IL-1, and IL-6, and reduces active remodeling units [60]. Moreover, estrogen inhibits bone loss by promoting apoptosis of osteoclasts by stimulating Fas ligand [61]. The increased NF-κB signaling in bone inhibits osteoblast differentiation and mineralization and promotes osteoclast proliferation. However, 17β-estradiol (E2) inhibits the NF-κB pathway, and E2 not only decreases osteoclast activation and bone resorption [62], but also induces osteoclast apoptosis [63,64]. In addition, estrogen-mediated osteoclast inhibition involves RANKL/OPG, where E2 increases the transcription of OPG, inhibiting RANKL binding to RANK, eventually, reducing osteoclast differentiation [64].

#### 2.2.2. Parathyroid Hormone (PTH)

PTH receptor (PTHR) signaling in osteoblasts and bone cells increases RANKL/OPG ratio and increases osteoclast recruitment and activity, thereby stimulating bone resorption [65]. The increasing level of osteoclast activating factor, parathyroid hormone-related peptide (PTHrP), induces bone resorption by upregulating RANKL receptor activators [66]. Continuous exposure to PTH activates bone resorption-related genes in osteoclasts. Because of the similar sequences in the amino-terminal region of PTH and PTHrP, PTH1R acts as a co-receptor of both, and through PTH1R, PTH and PTHrP promotes osteoclast formation and bone resorption [67]. PTH deficiency reduces RANKL and cathepsin K expression and affects PI3K/AKT/STAT5 pathway in osteoblasts, and suppresses osteoclast activity [68]. Moreover, deletion of cathepsin K promotes PTHrP and bovine PTH (bPTH) production. An increased concentration of bPTH enhances the resorption capability of osteoclasts by controlling intracellular acidification, the expression of vacuolar- H^+^-transporting adenosine triphosphatase (V-ATPase) subunits, and V-ATPase activity [69].

#### 2.2.3. Calcitonin

Calcitonin is released from the parafollicular cells in the thyroid gland, which rapidly reduces blood calcium and regulates calcium homeostasis by inhibiting bone resorption [70]. Calcitonin acts directly on osteoclasts via its receptor to reduce the motility and activity of osteoclasts [71]. The treatment of calcitonin on osteoclast inhibits bone resorption by disrupting sealing zones, dispersing V-ATPase, as well as reduces cell motility and cellular retraction through G proteins and cyclic AMP and calcium messengers [72,73]. CT also negatively regulates the expression of the *spns2* gene. This gene encodes a transporter for the signaling lipid sphingosine 1-phosphate (S1P), which couples bone formation to bone resorption [74].

### 2.3. V-ATPase

V-ATPase present in the ruffled membrane of osteoclast pumps proton into resorptive microenvironment to facilitate bone resorption by degrading bone matrix during bone resorption [75]. V-ATPase is made up of two parts: V*1* is an extrinsic catalytic part composed of eight subunits (A3, B3, C1, D1, E3, F1, G3, and H1) and V*0*, an intrinsic section, is composed of six subunits (a1, d1, e, c, c′, and c″). V-ATPase is responsible for releasing protons into the lumen of organelles using ATP [76] to mediate extracellular acidification of osteoclasts and bone resorption [77]. An atomic model of V-ATPases from the brain has revealed that the two type-I transmembrane proteins, Ac45 and a (pro)renin receptor, ATP6AP2/PRR along with subunit c”, are enclosed by c ring. Moreover, domains are also provided by c ring for cleaved ATP6AP1/Ac45 and ATP6AP2/PRR to facilitate the assembly of catalytic and membrane regions. The different conformations of subunits A/B cause rotation of subunits DF and stimulate d subunit to rotate with respect to the entire c-ring and help in V1-Vo rotation to transport protons [78,79]. Studies on mice and humans have shown that the V-ATPase subunits play a vital role in osteoclast-related diseases, including osteopetrosis and osteoporosis, and thus, can be a promising target for the development of anti-resorptive agents [80].

### 2.4. Non-Coding RNA

#### 2.5.1. MicroRNA (MiRNA)

MiRNAs constitute a class of single-stranded non-coding RNAs having a length of 19–25 nucleotides and are produced by the action of RNA polymerase II. MiRNAs regulate gene expression by preventing messenger RNA (mRNA) from being translated or promoting mRNA degradation, affecting cellular processes [81].

MiRNA regulates bone metabolism by regulating osteoblast and osteoclast differentiation. (Table 1) [82]. MiR-125a-5p promotes osteoclastogenesis by targeting the inhibition of TNFRSF1B [83], while miR-34c promotes osteoclast differentiation by targeting LGR4 to regulate NF-κB and glycogen synthase kinase 3-β signaling [84]. BCL-2-modifying factor (BMF) acts as a proapoptotic factor in osteoclasts. MiR-29b targets BMF and increases the number of osteoclasts without affecting osteoclast differentiation [85]. MiR-363-3p and miR-140-3p targets phosphatase and tensin homolog (PTEN) gene to activate the PI3K/AKT signaling pathway and promotes osteoclastogenesis, and inhibit osteoblast differentiation [86,87]. It was demonstrated that in osteoclast-specific miR-214 transgenic mice, PTEN levels were reduced, while osteoclast activity was increased. This increased osteoclast activity led to reduced bone mineral density in vivo [88]. Moreover, miR-142-5p also targets PTEN to promote osteoclastogenesis of BMMs through PI3k/Akt/FoxO1 pathway [89]. MITF, a key transcription factor involved in osteoclast differentiation, is inhibited by miR-340 [90]. One of the highly expressed miRNAs during osteoclast development upon RANKL stimulation is miR-31. Through inhibition experiment, it was found that miR-31 suppression inhibits actin ring formation, RANKL-induced osteoclast formation, and bone resorption, while RhoA expression, one of the miR-31 target genes, was increased. But these processes can be restored using a RhoA inhibitor [91]. Moreover, miR-214-3p in osteoclasts is associated with reduced bone formation in aging women with fractures and OVX mice. It has demonstrated that osteoclast-specific miR-214-3p knock-in in mice display an elevated level of exosomal miR-214-3p in serum and reduces bone formation. Importantly, transfer of exosomal miR-214-3p derived from osteoclast to osteoblasts reduces osteoblast activity in vitro and bone formation in vivo. However, miR-214-3p inhibition induces bone formation in aging OVX mice [92].

#### 2.5.2. Long Non-Coding RNA (LncRNA)

LncRNAs are non-coding RNA transcripts that constitute more than 200 nucleotides and regulate target gene expression via cis- or transregulation. [93]. It has been reported that the overexpression of lncRNA AK077216 during osteoclastogenesis promotes osteoclast differentiation and bone resorption by suppressing NIP45 and promoting NFATc1 expression [94]. Similarly, upregulated expression of lncRNA-MIRG in osteoclasts stimulates osteoclastogenesis and bone resorption by targeting miR-1897, which binds with NFATc1 and lowers its expression [95]. Additionally, lncRNA-Jak3 is known to activate and promote the expression of CTSK and NFATc1, which are well-known factors involved in osteoclastogenesis [96]. However, delta-like 3 (DLL3) targeting lncRNA LINC00311 inhibits osteoclast apoptosis and induces osteoclast proliferation via the Notch signaling pathway [97], whereas lncRNA CRNDE can promote osteoclast proliferation through PI3K/AKT [98]. LncRNA-MALAT1 negatively regulates miR-124, whose overexpression can reverse BMM migration and osteoclast differentiation, to enhance the recruitment and differentiation of osteoclast precursors [99].

#### 2.5.3. Circular RNA (CircRNA)

Circular RNA is a group of endogenous ncRNA that is covalently closed loop structure considered by-products of abnormal splicing. CircRNA lacks a 5′ or 3′ end which provides them the ability to resist exonuclease digestion. CircRNA regulates cellular transcription, posttranslational expression, by interacting with miRNAs. CircRNAs are directly involved in bone-related signal transduction, forming a circRNA-miRNA-mRNA axis to work in bone remodeling [93,100]. During RANKL + CSF1-induced BMM differentiation to osteoclast, circRNA_28313 level was elevated. The circRNA_28313 knockdown experiment demonstrated significant reductions in osteoclasts differentiation in vitro, and importantly, inhibited OVX-induced bone resorption in vivo. CircRNA_28313 targeted miR-195a, which inhibits CSF1 to promote osteoclast differentiation [101]. Another study reported that during osteoclast differentiation, the level of circRNA_009934 was also increased, and circRNA_009934 functioned as a ceRNA of miR-5107 to promote osteoclastogenesis [102]. Conversely, the overexpression of circ_0007059 reduced hBMSC differentiation into osteoclasts and bone morphogenetic protein 2 (BMP-2) in vitro by directly targeting miR-378 [103]. Liu et al. have demonstrated that circHmbox1 could also suppress RANKL-induced osteoclasts differentiation by binding to miR-1247-5p [104]. 

## 3. The Role of Osteoclasts in Bone Resorption

Osteoclast-mediated bone resorption involves multiple processes, including attachment to bone, re-organization of the cytoskeleton, formation of the ruffled border, and osteoclast polarization [105]. Once osteoclast is polarized, three membrane domains, namely, a ruffled border, a sealing zone, and a functional secretory domain, start to form. During cytoskeleton re-organization, the actin cytoskeleton attaches itself to the sealing zone, which encloses a ruffled border [106]. The formation of a ruffled border is regulated by a calcium sensor protein, synaptotagmin VII, which regulates exocytosis and lysosome activity in osteoclasts. Lack of synaptotagmin VII prevents ruffled border formation in osteoclasts, inhibiting the secretion of cathepsin K [107]. Actin ring formation is an important process for bone resorption in the sealing zone, which consists of certain contractile proteins with the help of αvβ3 integrins and ensuring migration of osteoclasts [105]. The process of actin polymerization and actin ring formation is controlled by Wiscott–Aldrich syndrome protein (WASP), which is activated by PIP2 and Cdc42-GTP [108]. 

During polarization, V-ATPase interacts with actin through the N-terminal domain and concentrates to the ruffled membrane, secreting H^+^ [109] into an extracellular compartment, called resorption lacuna to increase the surface area of osteoclasts that are in contact with the bone. The acid and a mixture of proteases dissolve crystalline hydroxyapatite and organic matrix, respectively. The secretion of H^+^ is balanced by the movement of Cl^−^ and alkalinization is modified by HCO_3_^−^/Cl^−^ [105,110]. The degraded collagen and matrix components are transcytosed through a functional secretory domain, allowing osteoclasts to remove degraded products without affecting their tight attachment to the bone [106]. After bone resorption, osteoclasts form a bone pit. Osteoblast precursors are then recruited to the bone pits to complement bone loss [111]. Furthermore, osteoclasts provide signals to osteoblast lineage cells to develop into osteoblasts by various mechanisms, such as releasing growth factors from the resorbed matrix, expressing membrane-bound factors, and producing secreted proteins and microvesicles [112].

## 4. Osteoporosis

Osteoporosis is a systemic bone disorder hallmarked by low bone mass, deteriorated bone tissue microstructure, increased bone brittleness, and fracture occurrence. In most cases, increased bone resorption is the main reason for the increased bone loss [113,114]. Osteoporosis is a multifactorial disease affected by various factors, such as nutrition, age, mechanical stress, genetic factors, and other diseases. Primary osteoporosis resulting from the losses associated with aging and/or menopause and secondary osteoporosis resulting from hypercortisolism, alcohol abuse, or medication are the common forms of osteoporosis [115].

During the development of osteoporosis, the bone strength is weakened owing to the defects in trabecular microarchitecture and disproportionate bone remodeling rate [116], which subsequently results in a series of clinical symptoms, including pain, fracture, and deformity. Therefore, an early diagnosis and effective treatment of osteoporosis are imperative. The measurement of bone mineral density (BMD) is a common method of diagnosing osteoporosis using dual-energy X-ray absorptiometry (DXA) and quantitative computed tomography (QCT) [117]. In addition to BMD, the calculation of fracture risk is also an important method for osteoporosis diagnosis, which can be calculated using the Garvan fracture risk calculator, QFracture^®^, and fracture risk assessment tool (FRAX^®^). Of the above, FRAX is the most commonly used and is a computer algorithm that calculates the 10-year probability of a fracture [118].

### Osteoclast Targeted Osteoporosis Treatments

**Figure 2:**
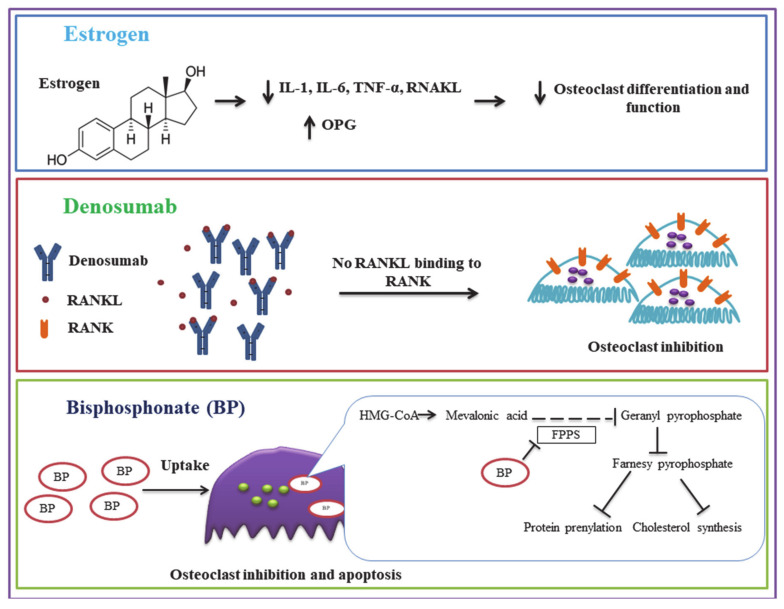
Pharmaceutical interventions for osteoporosis, targeting osteoclasts and their mechanism of action.

The pharmaceutical intervention (Figure 2) for osteoporosis mainly consists of two aspects: Bone formation stimulating agents and anti-resorptive agents [119]. Here we primarily present the anti-resorptive agents targeting osteoclasts. Bisphosphonates are the most common and typical anti-resorptive agents used for osteoporosis and are chemically stable structural analogs of inorganic pyrophosphate (PPi). Bisphosphonates adsorb to mineral surfaces and then are taken up by osteoclasts. Non-nitrogen-containing bisphosphonates, such as clodronate and etidronate, incorporate themselves into non-hydrolyzable adenosine triphosphate (ATP) analogs through class II aminoacyl–transfer RNA synthetases, preventing ATP-dependent intracellular enzymes. However, all nitrogen-containing bisphosphonates, including alendronate, inhibit farnesyl pyrophosphate synthase, an essential enzyme in the mevalonate pathway. As a result, the synthesis of cholesterol and isoprenoid compounds is inhibited, which are crucial for a posttranslational modification (isoprenylation) of Rab, Rho, and Rac. This disrupts the function of osteoclast, reducing bone resorption and osteoporosis [120]. Odanacatib, an inhibitor of cathepsin K, reduces the level of resorption markers and increases bone mineral density [121,122]. However, a clinical trial of odanacatib has reported an increased risk of stroke in osteoporosis postmenopausal women, and therefore, the development of odanacatib as an osteoporosis therapeutic was terminated [123]. Denosumab, a human monoclonal antibody against bone resorption mediator RANKL is used to reduce osteoclast-mediated bone resorption [124].

Similar mechanisms, by which endogenous hormones exert and control osteoclast activities, are utilized in hormone therapy [125]. In clinical studies, estrogen has been reported to promote bone mineral density at the lumbar spine and lower bone turnover, reducing the occurrence of vertebral fracture in postmenopausal osteoporosis women [126]. Moreover, selective estrogen receptor modulators (SERMs) are also used as anti-osteoporosis drugs. SERMs are non-steroidal compounds that act as agonists or antagonists to estrogen receptors (ERs) in a variety of tissues [127]. SERMs, such as raloxifene (an estrogen agonist) and bazedoxifene, alone or in combination with estrogen (tissue-selective estrogen complex (TSEC)) lower bone turnover and climacteric symptoms, maintaining and increasing vertebral and femoral BMDs [128]. Calcitonin is also used as a pharmacologic option for the treatment of osteoporosis. Calcitonin works by selectively disrupting actin ring, inducing the dispersion of V-ATPase and reducing pit-forming activity through PKA and protein kinase C (PKC)-mediated signals to inhibit OC function [129]. A randomized controlled trial showed that salmon calcitonin injection was significantly efficacious in postmenopausal osteoporosis in increasing BMD by stimulating osteoblast function and reducing bone absorption [130]. However, no significantly lowered incidences of fracture were detected [131].

An anti-fracture agent, strontium ranelate, is also used to treat postmenopausal osteoporosis, which actively promotes bone formation and inhibits bone resorption [132]. Strontium ranelate increases OPG and decreases RANKL expression to inhibit osteoclastogenesis and bone resorption [133]. A macrolide antibiotic bafilomycin A(1), SB-242784, and FR177995 selectively inhibit osteoclastic V-ATPases and bone resorption, preventing bone loss in rat models [134]. Despite the effective clinical effects of these medicines in alleviating osteoporosis, adverse effects, such as medication-related osteonecrosis of the jaw (MRONJ) are still reported. MRONJ, resulting from excessive usage of bisphosphonates and denosumab, is characterized by progressive deterioration of mandibular or maxillary bone [135]. Therefore, combination and alternative therapies have been identified as more promising strategies for osteoporosis improvement and management.

Factually, combination and alternative therapies, especially Traditional Chinese medicine (TCM), have been widely used for numerous chronic diseases worldwide. TCM is a holistic medical system with thousands of years of clinical practice [136]. Since discovering a well-known TCM, artemisinin, many clinical trials involving TCM for diseases have been reported [137,138]. Of the many Chinese medicines, *Herba Epimedium, Rhizoma Drynariae*, and *Salvia Miltiorrhiza* constitute saikosaponins, linarin, echinacoside, poncirin, and sweroside, which have the abilities to decrease the expression of osteoclast related genes and enhance osteoblast-associated gene expression [139]. Besides, *Salvia miltiorrhiza* and its constituents have been demonstrated to prevent osteoclast formation by reducing the expression of c-Fos and NFATc1, osteoclast differentiation by inhibiting RANKL expression and NF-κB induction, and block AKT, NF-κB, and MAPK signal transduction pathways to reduce osteoclast activity and number [139]. Polysaccharides from *Polygonatum sibiricum* can also reduce RANKL-activated osteoclastogenesis by enhancing β-catenin enrichment in the nucleus to lower the expression of osteoclast-related genes, and through the Hippo signaling pathway [136].

Many studies have successfully developed gene-based delivery systems to lower mRNAs, microRNA expression in osteoclasts. Many non-coding RNAs, such as miRNAs [81], lncRNAs, and circRNAs, have been reported to be implicated in osteoclast regulation during osteoporosis. These non-coding RNAs have been targeted through designing gene delivery systems functionalized with either osteoclast or bone-resorption surface targeting peptides, such as D-Asp_8_ and (Asp)_14_ or (AspSerSer)_6_, to reduce osteoclast-mediated bone resorption. The studies in animal models have shown promising results, demonstrating improved trabecular architecture and alleviated osteoporosis [140,141,142].

## 5. Conclusions

Bone remodeling plays an important role in maintaining bone homeostasis and bone physiology. Many cells, such as osteocytes, osteoblasts, and osteoclasts, play vital roles in bone remodeling. Osteoblast-mediated bone formation and osteoclast-mediated bone resorption are two important processes in bone remodeling. Osteoblast-secreted factors regulate the proliferation, differentiation, and function of osteoclasts. With aging and/or lack of regulatory factors, the function of osteoclast is increased, enhancing the rate of bone resorption that ultimately leads to bone disorders, including osteoporosis. The study of such factors in osteoclast regulations has paved the way for developing different therapeutics to regulate osteoclast-mediated bone resorption. However, available anti-resorptive therapeutics either have side effects or are in preclinical stages. Therefore, this area needs to be explored for developing safer and efficient therapeutics for osteoporosis.

In conclusion, osteoclasts play an important role in bone metabolism, and many proteins, hormones, and RNAs significantly affect the fate and function of osteoclasts. Therefore, developing therapeutics with the potential to target these factors can help in controlling the function of osteoclasts to alleviate bone diseases.

## Figures and Tables

**Table 1:** MiRNAs that are involved in osteoclast regulation.

MiRNA	Role	Target	Reference
MiR-125a-5p	Promotes osteoclastogenesis	TNFRSF1B	[83]
MiR-34c	Promotes osteoclast differentiation	LGR4	[84]
MiR-29b	Increases osteoclasts number	BMF	[85]
MiR-363-3p	Promote osteoclastogenesis	PTEN	[86]
MiR-140-3p			[87]
MiR-214			[88]
MiR-142-5p			[89]
MiR-340	Inhibits osteoclast differentiation	MITF	[90]
MiR-31	Regulates osteoclast cytoskeleton organization	RhoA	[91]
MiR-214-3p	Reduces bone formation	ND	[92]

ND—Not disclosed.

## Data Availability

Not applicable.
